# Noninvasive Monitoring during Interhospital Transport of Newborn Infants

**DOI:** 10.1155/2013/632474

**Published:** 2013-02-24

**Authors:** Georg M. Schmölzer, Megan O'Reilly, Po-Yin Cheung

**Affiliations:** ^1^Department of Pediatrics, University of Alberta, Edmonton, AB, Canada 11405-87; ^2^Neonatal Research Unit, Royal Alexandra Hospital, 10240 Kingsway Avenue NW, Edmonton, AB, Canada T5H 3V9; ^3^Division of Neonatology, Department of Pediatrics, Medical University of Graz, 8010 Graz, Austria

## Abstract

The main indications for interhospital neonatal transports are radiographic studies (e.g., magnet resonance imaging) and surgical interventions. Specialized neonatal transport teams need to be skilled in patient care, communication, and equipment management and extensively trained in resuscitation, stabilization, and transport of critically ill infants. However, there is increasing evidence that clinical assessment of heart rate, color, or chest wall movements is imprecise and can be misleading even in experienced hands. The aim of the paper was to review the current evidence on clinical monitoring equipment during interhospital neonatal transport.

## 1. Introduction

The main indications for interhospital neonatal transports are radiographic studies (e.g., magnet resonance imaging) and surgical interventions. Specialized neonatal transport teams need to be skilled in patient care, communication, and equipment management and extensively trained in resuscitation, stabilization, and transport of critically ill infants [1]. Clinical monitoring equipment routinely used in the neonatal intensive care unit (NICU) may not function optimally under transport conditions [2]. Both the critically ill neonate and the neonatal transport team are exposed to mechanical stressors (e.g., shock, vibration, and noise) making clinical assessment during transport almost impossible [1, 3–9]. However, most of the equipment routinely used in the NICU to support clinical management decisions has not been evaluated in the transport environment. The aim of the paper was to review the current evidence on clinical monitoring equipment during interhospital neonatal transport.

## 2. Search Strategies

The aim of this paper is to review the available literature about monitoring during interhospital neonatal transport. We reviewed books, resuscitation manuals, and articles from 1950 to the present with the search terms “infant,” “newborn,” “neonatal transport,” “pulse oximetry,” “heart rate,” “respiratory function tests,” “carbon dioxide,” “temperature,” “blood pressure monitoring” and “transport scores.” The full search strategy for PubMed is detailed in the Appendix.

## 3. Pulse Oximetry and Heart Rate 

Neonatal transports carried out overnight make the assessment of an infant's color challenging. In addition, incubators are covered to decrease environmental impact, which blocks light inside the incubator making color assessment challenging. Furthermore, judging an infants color to determine oxygen saturation is imprecise [10]. During neonatal transport, vibration can cause intermittent failure or signal artifacts [6, 11, 12]. Short et al. tested seven different pulse oximeters during helicopter flights, and the majority of pulse oximeters demonstrated minimal signal artifacts [6]. 

HR is the most important clinical indicator of adequate breathing and respiratory support [13, 14]. International resuscitation guidelines recommend to assess an infant's HR during neonatal resuscitation using a stethoscope [14]. However, an observational delivery room study showed that auscultation is inaccurate and underestimates HR compared to HR measurements using electrocardiogram [15, 16]. Kamlin et al. demonstrated that HR displayed by pulse oximetry is as accurate as HR obtained by a 3-lead electrocardiogram, including those newborns receiving advanced resuscitation [17]. Hence, pulse oximetry can be used to monitor an infant's HR during neonatal transport. In addition, the HR is displayed continuously allowing the team to continue any neonatal transport without stopping to listen to the HR.

In summary, pulse oximetry should be the standard of care for managing infants during neonatal transport, enabling immediate and dynamic assessment of oxygenation and heart rate.

## 4. Noninvasive Blood Pressure Monitoring

Norm values of BP in newborn infants are derived from a reference population with regard to gestational age, birth weight, and postnatal age [18–20]. Overall, different definitions are used to diagnose neonatal hypotension: (i) BP <10th percentile of normative blood pressure values, (ii) mean arterial blood pressure equals gestational age in whole weeks and no other signs of hypoperfusion (e.g., high serum lactate concentration or oliguria) exist. This definition can only be used within the first 5 days after birth as mean arterial blood pressure increases up to 10 mmHg during this time, and (iii) mean arterial blood pressure <30 mmHg, which is based on the assumption that cerebral blood flow becomes pressure dependent at a mean arterial blood pressure around 30 mmHg [18, 19]. The goal of blood pressure (BP) monitoring is to optimize cardiac output, which is generally relied on by clinical assessment, HR, and BP monitoring. Therefore, adequate BP does not always indicate adequate cardiac output. Continuous arterial BP monitoring using an indwelling catheter is considered the “gold standard” of BP measurement in the critically ill neonate [18, 19]. Although BP is also frequently noninvasively measured, it is less accurate (especially in severe hypotension) and not continuous. Most importantly noninvasive BP measurements are not continuous, inaccurate (overestimation of hypotension and underestimation of hypertension), and unable to provide reliable mean or diastolic arterial BP. However, they can be used for trends in BP changes [18, 19]. Noninvasive BP measurements should be available for all transported newborns, however automated non-invasive BP devices can be affected by vibration and motion. In addition, various cuff sizes are needed as a too small cuff overestimates blood pressure. 

## 5. Respiratory Function Monitor (RFM)

During neonatal resuscitation, mask ventilation should be guided by observing chest rise [14, 21]. However, recent observational studies in the delivery room have demonstrated that observation of chest rise movements to assess positive pressure ventilation is imprecise [22, 23]. A similar thing can be said during neonatal transport, where assessment of chest rise to assess ventilation is limited. In comparison, a respiratory function monitor (RFM) provides objective measurements of continuously measured respiratory parameters [1, 24, 25].

In comparison, guidance of mechanically ventilated newborn infants by displayed respiratory function is a standard of care in the NICU [26]. In addition, tidal volume monitoring has recently been advocated for neonatal resuscitation and neonatal simulation [23–25, 27–29]. However, this technique has not been implemented during neonatal transport. During neonatal transport, mechanical ventilation is indirectly assessed using HR, SpO_2_, chest rise, end-tidal CO_2_ (ETCO_2_) or transcutaneous CO_2_- (TCO_2_), and O_2_-tension [30–33]. Observational studies in the delivery room have demonstrated that chest rise is a poor proxy for tidal volume delivery regardless of the level of experience [22, 23]. Tracy et al. reported that 25% of preterm infants receiving positive pressure ventilation (PPV) while transported from the delivery room to the NICU had hypocapnia on arrival [34, 35]. They showed that by 20 minutes after birth, 20% of infants had a PaCO_2_ below 25 mm Hg—a known risk factor for brain injury [35, 36]. Lilley et al. reported similar results. Infants were more likely to be overventilated when clinical assessment was used to guide PPV during neonatal transport [37]. In comparison, when an RFM was used, target TCO_2_ tension was achieved within 15 minutes after PPV was started [37]. However, the study design and low numbers of included infants did not allow the results to be directly attributed to the use of the RFM.

**Figure 1:**
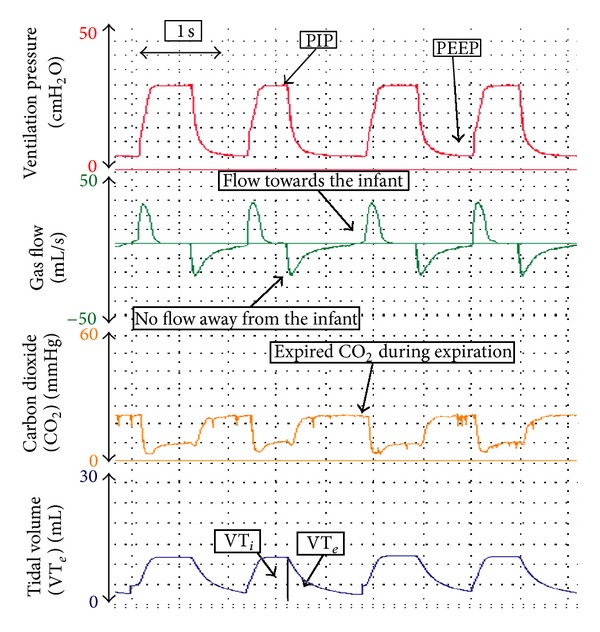
During positive pressure ventilation (PPV), the airway pressures rise to set PIP. At the end of inspiration, PIP decreases to baseline (PEEP). The area underneath the gas flow waves during inflation and expiration is similar, which is reflected in the *V*_*T*_ wave returning to the baseline after expiration. No leak is displayed.

**Figure 2:**
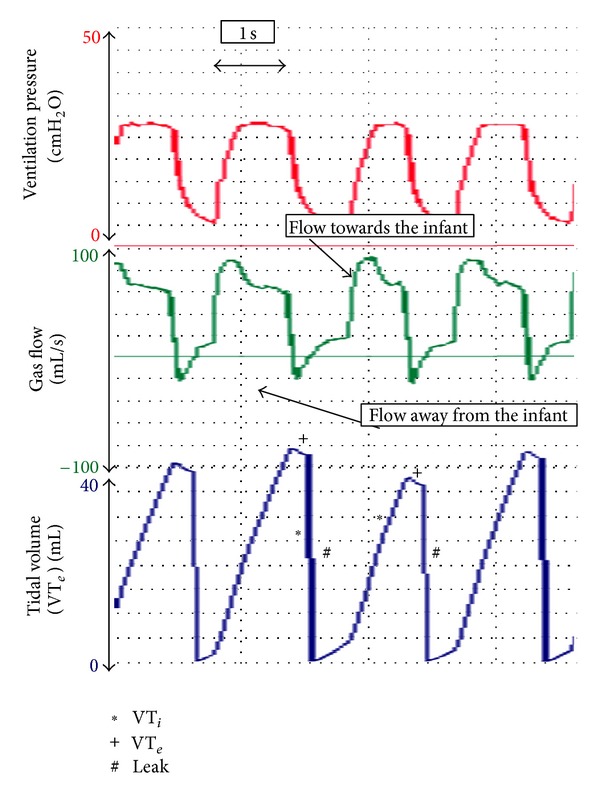
During PPV, the area underneath inspiratory gas flow is larger compared to expiratory gas flow. This is reflected in the display of a large amount of leak around the ETT.

**Figure 3:**
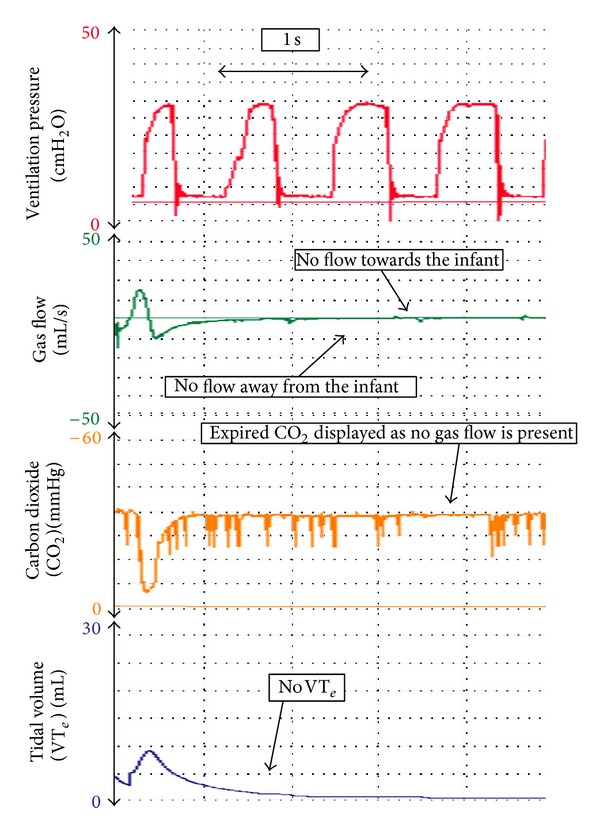
During PPV, the ETT suddenly becomes obstructed, which can be identified by gas flow and *V*_*T*_ cessation. Airway pressures are continuously delivered.

**Figure 4:**
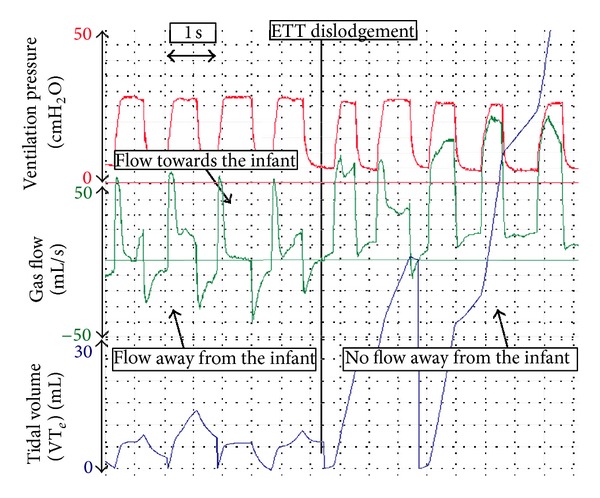
During PPV, the ETT suddenly becomes dislodged.

**Figure 5:**
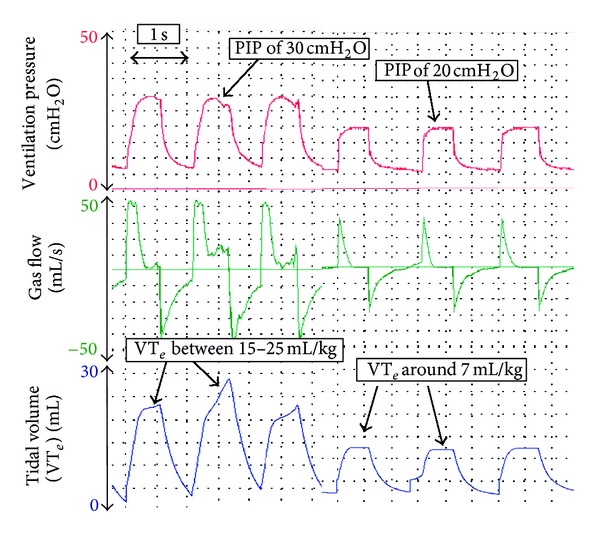
During PPV, the delivered *V*_*T*_ is between 21 and 30 mL/kg. Once the PIP is decreased from 30 cm H_2_O to 20 cm H_2_O, the displayed *V*_*T*_ decreases to around 9 mL/kg.

**Figure 6:**
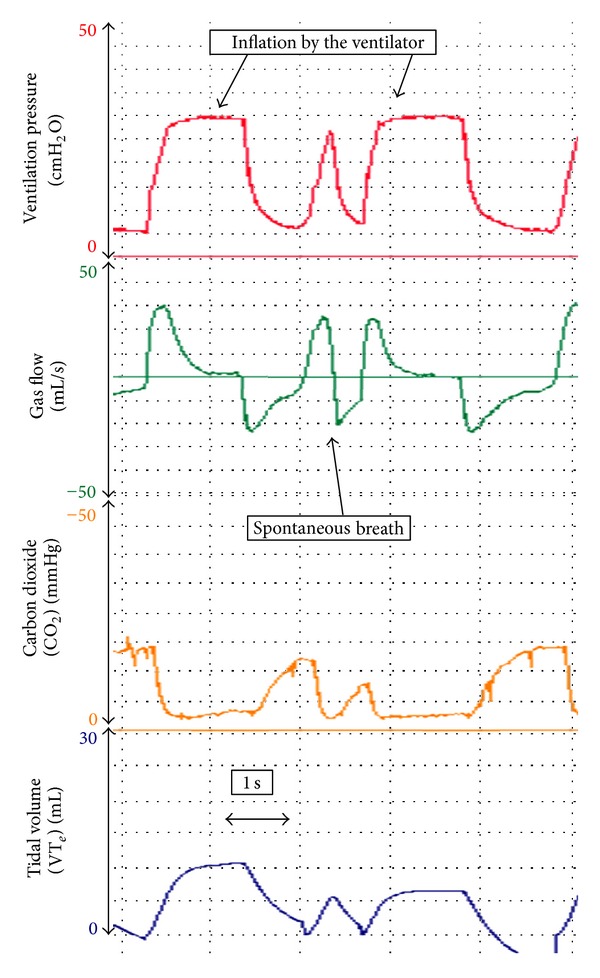
During manual inflations the infant is taking a spontaneous breath.

A respiratory function monitor (RFM) continuously displays graphical waveforms and numerical values of peak inflation pressure (PIP), positive end-expiratory pressure (PEEP), tidal volume (*V*_*T*_), leak around an endotracheal tube (ETT), minute ventilation, respiratory rate, and inspiration and expiration times (Figure 1) [1, 24, 25]. To measure respiratory function, a flow sensor is placed between the ventilator and an ETT; *V*_*T*_ is automatically calculated by integrating the flow signal, and leak around the ETT is displayed as a percentage of the inspired *V*_*T*_ [38]. Airway pressures (e.g., PIP and PEEP) are directly measured from the circuit. We believe that during neonatal transport, an RFM can be used to (i) identify leak around an ETT (Figure 2) [24, 39, 40], accidental extubation (Figure 3) [24, 39–41], airway obstruction (Figure 4) [24, 42], adequate *V*_*T*_ delivery (Figure 5) [22, 23, 27], and observing spontaneous breathing during mechanical ventilation (Figure 6) [24, 43–45].

### 5.1. Endotracheal Tube Size

An RFM can show the percentage of leak around the ETT. With an appropriate sized ETT, any leak will be small. However, if a too narrow ETT is inserted, a large leak of the inflating volume around the ETT will be displayed at any RFM (Figure 2). This means that insufficient gas enters the lung, which may result in unsatisfactory mechanical ventilation. The use of an RFM enables the transport team to observe the ETT leak continuously during every breath cycle and assess if the ETT size should be changed [24, 37]. 

### 5.2. Accidental Extubation

During neonatal transport, an ETT can easily become dislodged. This can be seen immediately on the flow and volume signals as there is little or no expiratory flow and volume (Figure 3) [24].

### 5.3. Airway Obstruction

If there is little or no increase in a low *V*_*T*_ wave in response to an increase in PIP, then airway obstruction should be considered [24]. Airway obstruction has been reported during mask ventilation [42, 46], after surfactant administration [47, 48], and blockage due to blood or secretion. Figure 4 demonstrates adequate PPV via an ETT. Suddenly, the ETT becomes obstructed, which is indicated by almost no gas flow and no *V*_*T*_ wave.

### 5.4. Tidal Volume Delivery

The purpose of applying a PIP during PPV is to inflate the lungs with an appropriate tidal *V*_*T*_ and thereby facilitate gas exchange [24]. When a fixed pressure is used, the delivered *V*_*T*_ will be dependent on the size of the infant, compliance of the lungs and chest wall, and resistance of the airways [24, 29, 49, 50]. Too high *V*_*T*_ delivery can cause lung injury by over inflation and hypocapnia, and too small *V*_*T*_ will result in inadequate gas exchange [15, 24, 29, 30, 35, 51–55]. The current evidence suggests that *V*_*T*_ should be within the range of 4 to 8 mL/kg [26, 29, 51]. Using an RFM during neonatal transport enables the transport team to adjust the set PIP to ensure adequate *V*_*T*_ is delivered (Figure 5). The optimum PIP will vary between infants and in the same infant over time depending of the cause of lung injury (e.g., congenital diaphragmatic hernia, lung hypoplasia, meconium aspiration, or bronchopulmonary dysplasia).

### 5.5. Observing Spontaneous Breathing

During mechanical ventilation, an RFM attached to an ETT can be used to display the spontaneous *V*_*T*_ and interactions between spontaneous breaths and inflations made by the ventilator (Figure 6) [24, 45, 56]. This can assist the neonatal team to determine if a ventilated newly born infant is apnoeic, or breathing synchronously or asynchronously with the manual inflations [24]. An RFM may show the infant “fighting the ventilator” and breathing completely out of phase with the mechanical inflations (e.g., infant inspires during mechanical expiration and expires during inflations), which is inefficient and potentially traumatic [24, 56, 57].

## 6. Carbon Dioxide Monitoring

Continuous noninvasive CO_2_ monitoring (e.g., ETCO_2_ or TCO_2_) has become an important bedside tool during neonatal transport [31, 33]. ETCO_2_ can be measured using either main-, side- or microstream technology [31, 33, 58]. Clinical applications for CO_2_ monitoring include (i) confirmation of correct tube placement and (ii) guidance of mechanical ventilation [31, 33, 39, 40].

### 6.1. Confirmation of Correct Tube Placement

Bhende et al. reported that ETCO_2_ can be used to assess correct ETT position during neonatal transport [33]. However, recent delivery room studies have demonstrated that ETCO_2_ monitoring failed to correctly identify ETT placement in up to one third of the cases [39, 59]. 

### 6.2. Guidance of Mechanical Ventilation

The gold standard for assessing the adequacy of mechanical ventilation is arterial blood gas analysis [60]. However, noninvasive CO_2_ monitoring has become an important bedside tool during neonatal transport [31, 33, 60, 61]. Several studies compared arterial CO_2_ with ETCO_2_ or TCO_2_ values [31, 33, 60, 61]. Tingay et al. compared ETCO_2_, TCO_2_, and arterial CO_2_ during neonatal transport [31]. They found no correlation between ETCO_2_ and arterial CO_2_ measurements [31], suggesting that TCO_2_ should currently be used during neonatal transport. Tobias summarized the available literature of TCO_2_ in infants and children [62]. When compared to ETCO_2_, TCO_2_ is as accurate in patients with normal respiratory function. In addition, it was more accurate in patients with congenital heart disease and right-to-left shunting [62]. However, ETCO_2_-monitoring remains the standard of care to demonstrate the correct ETT placement or ETT disconnection. Overall, there are several limitation to each technique [31, 33, 60, 61]. Although arterial blood gas analysis is the gold standard, the monitoring is not feasible particularly in the prehospital setting because of the lack of specialized equipment and expertise required for placement and monitoring [2]. Major concerns while using TCO_2_ include (i) vasodilatation of the capillary bed beneath the TCO_2_ probe, which might cause TCO_2_ value alterations, (ii) heating of the TCO_2_ probe to 43°C can cause burn injuries and increases tissue metabolic rate by 4-5% for every °C, (iii) improper calibration, trapped air bubbles, and damaged membranes are possible and may be difficult to detect, (iv) hyperxemia (PaO_2_ >100 torr), (v) shock or acidosis, (vi) or improper electrode placement might increase the discrepancy between arterial CO_2_ and TCO_2_ values [1, 60, 62, 63]. In comparison, birth weight, site of TCO_2_ probe, mean blood pressure and airway pressure do not affect TCO_2_ measurement [1, 60, 62, 63]. Although ETCO_2_ and TCO_2_ are promising the current available methods should only be used to complete arterial CO_2_ monitoring during neonatal transport.

## 7. Temperature

Maintaining the thermal environment for newborn infants and avoidance of cold stress is important for short- and long-term outcomes [64]. Rates of hypothermia (<36%) decreased over the last decades during neonatal transport [3, 5, 65]. However, one third of the infants ≤1000 g had hypothermia at arrival of the transport team, and remained hypothermic despite active warming [65]. In comparison, a significant increase in hyperthermia from 12% in 1977–79 to 24% in 1995-96 has been observed for all infants except for infants ≤1000 g [65].

Recently whole-body cooling for hypoxic ischemic encephalopathy has been introduced during neonatal transport. Whole-body cooling can be achieved by using either passive (naturally cooling with no external intervention) or active (e.g., cold gel-packs) cooling [66–70]. Both methods have the potential for both over- and undercooling particularly without appropriate monitoring [67–70].

The optimal method to monitor body temperature during whole-body cooling remains controversial. Esophageal temperature monitoring has been reported to be more accurate compared to measurements obtained from tympanic, rectal, axillary, or the bladder [70]. In addition, skin probes rely on skin perfusion and are unreliable during whole-body cooling [70]. Currently, rectal probes should be used to continuously monitor body temperature during therapeutic hypothermia.

## 8. Neonatal Scores

Different neonatal scores are used to assess newborn infants [71–77]. The transport risk index of physiologic stability (TRIPS) (temperature, BP, respiratory distress/pulse oximetry, and response to noxious stimuli) assesses an infant before and after transport, and change is detected by comparing pre- and posttransport scores [71, 74]. A higher total TRIPS score indicates more severely ill newborn, and a higher posttransport score has been associated with increased neonatal mortality and intraventricular hemorrhage. The score for neonatal acute physiology II (SNAP-II) (mean BP, temperature, PO_2_/FiO_2_ ratio, serum pH, seizures, and urine output) and SNAP-perinatal extension-II (SNAPPE-II) (additionally to SNAP includes birth weight, 5-minute Apgar score, and small for gestational age) are illness severity and mortality risk scores for newborns in the NICU [71–77]. SNAP-II has been validated as a measure of newborn illness severity and SNAPPE-II as a measure of mortality risk however; both scores were not originally designed to assess interhospital transport [76]. Two studies compared the scores for infants receiving neonatal transport. Lee et al. did not find a significant difference between TRIPS and SNAP-II in their ability to predict mortality and severe intraventricular hemorrhage [74]. In comparison, Lucas de Silva et al. reported that TRIPS scores at admission were able to predict one week mortality in preterm infants <32-weeks gestation [77].

In summary, TRIPS score calculated at admission is predictive of early neonatal mortality in infants with <32-week gestation. TRIPS might be a useful triage tool if applied at the time of first contact with a transport service.

## 9. Conclusion

The information presented in this paper is from applicable animal and clinical studies during neonatal transport, the NICU, and during delivery room resuscitation. Unfortunately, there is a lack of data and randomized studies during neonatal transport, which are urgently needed. However, it is extremely difficult to undertake good detailed randomized studies during emergency neonatal transports. We leave it to the reader to interpret the results of the presented studies. However, any neonatal transport team requires skills in patient care and equipment management. In addition, advanced training in neonatal resuscitation, stabilization, and transport of these infants are pinnacle.

## Appendix

### Search Strategy for PubMed

(Last search 01/01/2013): limits activated (human)

#1 MeSH Term “Infant” (result: 847,904)
#2 MeSH Term “Newborn” (result: 470,261)
#3 Keyword “Neonatal transport” (result: 1,873)
#4 ((#1) AND #2) AND #3 (result: 1,247)
#5 MeSH Term “Pulse Oximetry” (result: 11,191)
#6 MeSH Term “Heart Rate” (result: 171,586)
#7 MeSH Term “Respiratory Function” Tests (result: 160,697)
#8 MeSH Term “Carbon Dioxide” (result: 39,322)
#9 MeSH Term “Temperature” (result: 149,958)
#10 MeSH Term “Blood Pressure monitoring” (result: 28,404)
#11 Keyword “Transport Scores”
#12 (#4) AND #5 (result: 22)
#13 (#4) AND #6 (result: 29)
#14 (#4) AND #7 (result: 176)
#15 (#4) AND #8 (result: 46)
#16 (#4) AND #9 (result: 79)
#17 (#4) AND #10 (result: 13)
#18 (#4) AND #11 (result: 35)

## Abbreviations

NICU: Neonatal intensive care unit
SpO_2_: Oxygen saturation
HR: Heart rate
BP: Blood pressure
RFM: Respiratory function monitor
ETT: Endotracheal tube
PIP: Peak inflation pressure
PEEP: Positive end-expiratory pressure
*V*_*T*_: Tidal volume
CO_2_: Carbon dioxide
ETCO_2_: End tidal carbon dioxide
TCO_2_: Transcutaneous carbon dioxide
PPV: Positive pressure ventilation
TRIPS: Transport risk index of physiologic stability
SNAP-II: Score for neonatal acute physiology II
SNAPPE-II: SNAP-perinatal extension-II.
